# Geographical patterns and environmental influencing factors of variations in *Asterothamnus centraliasiaticus* seed traits on Qinghai-Tibetan plateau

**DOI:** 10.3389/fpls.2024.1366512

**Published:** 2024-03-28

**Authors:** ZhengSheng Li, YuShou Ma, Ying Liu, YanLong Wang, XinYou Wang

**Affiliations:** Qinghai University, Qinghai Academy of Animal and Veterinary Sciences, Qinghai Provincial Key Laboratory of Adaptive Management on Alpine Grassland, Key Laboratory of Superior Forage Germplasm in the Qinghai-Tibetan Plateau, Xining, Qinghai, China

**Keywords:** *asterothamnus centraliasiaticus*, environmental influencing factor, geographic variation, seed trait, Qinghai-Tibetan plateau

## Abstract

**Introduction:**

Seed traits related to recruitment directly affect plant fitness and persistence. Understanding the key patterns and influencing factors of seed trait variations is conducive to assessing plant colonization and habitat selection. However, the variation patterns of the critical seed traits of shrub species are usually underrepresented and disregarded despite their vital role in alpine desert ecosystems.

**Methods:**

This study gathered seeds from 21 *Asterothamnus centraliasiaticus* populations across the Qinghai-Tibetan Plateau, analyzing geographical patterns of seed traits to identify external environmental influences. Additionally, it explored how seed morphology and nutrients affect germination stress tolerance, elucidating direct and indirect factors shaping seed trait variations.

**Results:**

The results present substantial intraspecific variations in the seed traits of *A. centraliasiaticus.* Seed traits except seed length-to-width ratio (LWR) all vary significantly with geographic gradients. In addition, the direct and indirect effects of climatic variables and soil nutrients on seed traits were verified in this study. Climate mainly influences seed nutrients, and soil nutrients significantly affect seed morphology and seed nutrients. Furthermore, climate directly impacts seed germination drought tolerance index (GDTI) and germination saline-alkali tolerance index (GSTI). Seed germination cold tolerance index (GCTI) is influenced by climate and soil nutrients (mostly SOC). GDTI and GSTI are prominently influenced by seed morphology (largely the seed thousand-grain weight (TGW)), and GCTI is evidently affected by seed nutrients (mainly the content of soluble protein (CSP)).

**Discussion:**

The findings of this study amply explain seed trait variation patterns of shrubs in alpine desert ecosystems, possessing significant importance for understanding the mechanism of shrub adaptation to alpine desert ecosystems, predicting the outcomes of environmental change, and informing conservation efforts. This study can be a valuable reference for managing alpine desert ecosystems on the Qinghai-Tibetan Plateau.

## 1 Introduction

Seeds are not only the reproductive organs of plants but also living organisms capable of independent development, which have evolved over time in nature (William and Gerhard, 2006). Seed traits are closely related to plant growth processes, partly reflecting the stably inherited original information of plants (Hiroyuki, 2014). Furthermore, as a resource input from the plant to future generations, seeds are involved in plant colonization, habitat selection, and the consequent renewal of species communities (Chauhan and Johnson, 2010; Anne et al., 2015). It is well established that variations in seed traits are an evolutionary behavior of plants responding to the selection and inheritance in their living environments (Alejandra and Luciana, 2015). Seed trait variations also represent the plasticity of seed morphological traits, which is the trade-off strategy of plants under environmental change (Victor, 2007). Therefore, exploring the variation patterns of seed traits and influencing factors can facilitate the comprehension of plant adaptation mechanisms, the prediction of environmental change outcomes, and advancements in conservation efforts (Anne et al., 2015).

The variability of seed traits across geographic gradients has undergone thorough investigations. A consistent change in seed morphology from the equator to the poles has been reported, particularly seed mass decline (Angela and Mark, 2004; Pieter et al., 2013). It is assumed that higher temperatures and stronger solar radiation at lower latitudes favor plants generating more photosynthetic products and correspondingly larger seeds (Murray et al., 2004). Nevertheless, some studies suggest that seed traits do not regularly vary with latitude (Alice and Katherine, 1993). Wu et al. (2018) described a significant decrease in seed morphology with longitude, including seed length, width, and thousand-seed weight. However, the current understanding of the relationship between seed trait variations and longitude is limited to the negative correlation of seed morphology with longitude, which is attributed to the effect of mean annual precipitation (MAP), an incremental vertical humidity effect from the ocean to the inland (Wu et al., 2018). A comprehensive and definitive explanation for the variations in seed traits with longitude, including seed morphology, seed nutrients, and seed germination stress tolerance indexes, remains elusive. Seeds are believed to be smaller and more elongated, with a larger surface area at higher altitudes (Bu et al., 2007; Qi et al., 2014). Some studies believe that this greater surface area is conducive to faster germination, highlighting the direct influence of seed morphology on germination (Ruprecht et al., 2015; Wang et al., 2021). However, the veracity of these claims is still under debate. Also in urgent need of clarification is whether seed trait variations are influenced by other environmental factors.

Research on plant shade tolerance strategies suggests an inconspicuous relationship between seed traits (represented by seed mass) and soil nutrients (Reich et al., 1998). Conversely, experimental results of Jager et al. (2015) significantly deviate from it. This underlines the importance of considering soil factors besides climatic elements in the environment where the parent plant thrives. Soil attributes, such as pH, organic carbon (SOC), and available nitrogen (AN), can contribute to seed trait variations in varying degrees (Malhi et al., 2014; Huang et al., 2016).

Most studies agree that the effects of soil nutrients on seed traits are mainly reflected in the variability of seed nutrients (Fenando et al., 2000). These nutrient variations further influence seed morphological traits, represented by seed mass (Wu et al., 2018). Zhang et al. (2016) concluded that differences in the content of AN in the soil significantly affected the composition of amino acids in the seed (Mon et al., 2016), further promoting the overall content of soluble proteins (CSP) in the seed (Zhu et al., 2018). Additionally, high levels of nitrogen lead to a decrease in the content of crude fat (CCF) in the seed and an opposite trend in CSP and CCF (Urbaniak et al., 2008; Solis et al., 2013). Some reports claim that seed fat content depends more on the content of SOC (Onemli, 2014). Comprehensive studies on soil available phosphorus (AP) and its impact on seed trait variations are insufficient, with few consensuses on this aspect. It is generally believed that the combined action of nitrogen and phosphorus can lead to changes in plant seed nutrients (Uddin et al., 2014). A recent study by Wu et al. (2018) on the driving factors of seed trait variations in a relict tree species discovered that soil phosphorus determined the geographic variation of seed traits under various soil nutrient conditions. In summary, the effect of soil nutrients on seed traits may vary with geography and species. However, the understanding of these effects is inadequate.

As the primary organ of plants, seeds store nutrients for germination and the growth of the next generation. Therefore, they must maintain adaptability to adversity within their propagation range (Li and Liu, 2023). It has been suggested that environmental conditions indirectly affect seed germination by influencing the amount and types of nutrients transferred from the parent plant to the seed (Donohue, 2009). This nutrient transference is considered one of the strategies that seeds use to cope with adversity and ensure successful germination (Li et al., 2017). Soluble protein, soluble sugar, starch, and crude fat are fundamental nutrients in the transference (Hong et al., 2012), playing a crucial role in seed germination before plant photosynthetic autotrophy (Delefosse et al., 2016; Benincasa et al., 2019). However, there are divergences in the influencing mechanism of these seed nutrients on germination. It is extensively believed that a positive correlation exists between the percentage and speed of seed germination and the CSP, content of soluble sugar (CSS), and starch (CS), and a negative correlation with CCF (Singkhornart and Ryu, 2011; Saatkamp et al., 2019). Nevertheless, the opposite conclusion has also been proposed, suggesting that high soluble protein content may be detrimental to seed germination (LeVan et al., 2008).

As mentioned earlier, the mobilization of stored substances during seed germination may vary with their content and species. Unfortunately, unanimous conclusions on the relationship between seed intrinsic attributes and germination are rare. Although some findings have been achieved, they mostly pertain to cash crops and model plants (Kesavan et al., 2013). Moreover, seed stress tolerance during germination has received even less attention. It is particularly challenging to generalize this relationship to wild shrub species, which are generally neglected irrespective of their prominent role in alpine desert ecosystems.

Characterized by complex and variable microhabitats and a wide range of species diversity, the Qinghai-Tibetan Plateau is ideal for investigating geographical patterns and environment-driven mechanisms of seed trait variations (Cui et al., 2015). *Asterothamnus centraliasiaticus* Novopokr., a dominant community-building species of the Qinghai-Tibet Plateau, is indispensable, contributing to soil stabilization, material cycling, and energy flow in the alpine desert ecosystem (Li et al., 2023). Given these attributes, empirical studies linking intraspecific plant variations to the environmental conditions of *A. centraliasiaticus* are of profound importance, facilitating the recognition of shrub adaptation in alpine desert ecosystems, the forecast of the outcomes of environmental change, and conservation efforts.

This study collected seeds of *A. centraliasiaticus* from 21 populations across its natural distribution on the Qinghai-Tibetan Plateau. Geographical patterns of seed traits were investigated to explore external environmental influencing factors of seed trait variations. Additionally, this study emphasized the influence of seed intrinsic attributes on germination stress tolerance capabilities through seed morphology and nutrients to ultimately clarify direct and indirect influencing factors of seed trait variations. Specifically, the following questions were focused on: (1) Are there substantial variations in seed traits among alpine desert shrub populations? If so, do seed traits vary significantly along geographic gradients? (2) Among multiple environmental factors, which one dominantly impacts seed trait variations? (3) Are the mechanisms affecting seed germination drought, cold, and saline tolerance the same?

## 2 Materials and methods

### 2.1 Research area

The study area covered nearly the entire geographic distribution of *A. centraliasiaticus* on the Qinghai-Tibetan Plateau (35.83-38.32°N and 90.86-102.76°E), which was attributed to this study’s previous systematic field investigation on this area and the accurate simulation of the geographic distribution of *A. centraliasiaticus* using the Maxent model. It spanned ca. 251 km along latitude and ca. 1071 km along longitude. The altitude of the study area ranged from 1,866 to 3,832 m a.s.l. The region has a plateau continental climate typified by aridity, with a MAP decreasing from 427 mm in the east to 67 mm in the west and a MAT between -1.5°C and 8.3°C. The vegetation in this region is dominated by drought-tolerant and salt-tolerant shrubs, including *A. centraliasiaticus*, *Reaumuria songarica*, *Krascheninnikovia ceratoides*, *Haloxylon ammodendron*, *Corethrodendron multijugum, Ajania fruticulosa, Brachanthemum pulvinatum, Kalidium foliatum*, and *Artemisia gyangzeensis*. It also contains patches of drier herb vegetation, such as *Leymus secalinus* and *Orinus kokonorica*.

### 2.2 Seed collection

**Figure 1:**
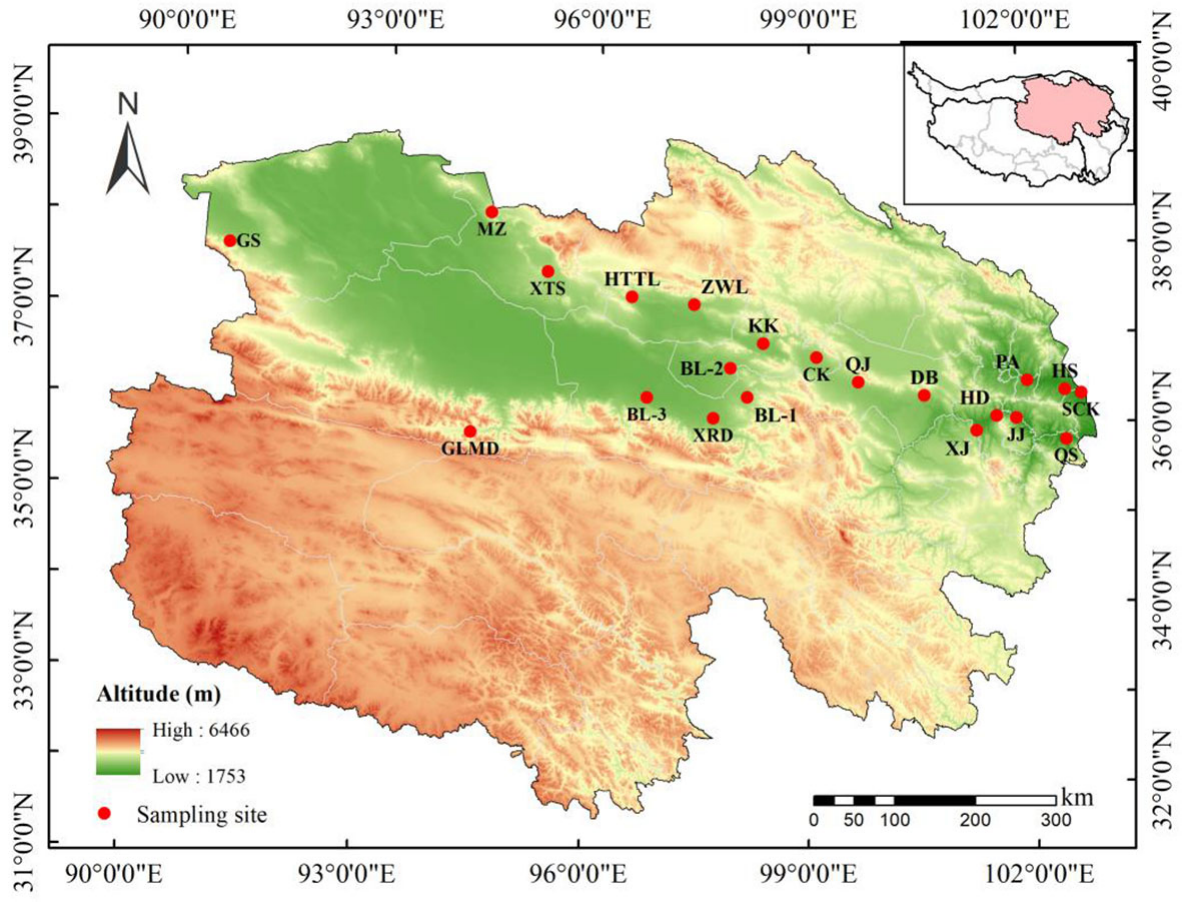
Locations of the 21 populations of *Asterothamnus centraliasiaticus* across its geographic distribution on the Qinghai-Tibetan Plateau. See **Table A1** for the site code.

Seeds of *A. centraliasiaticus* were collected at 21 sampling sites from August to September 2023 (**Figure 1**; **Table A1**). This study randomly searched three populations of *A. centraliasiaticus* for three sampling replicates within 2 km around the sampling sites as much as possible. These test replicates of each population are regarded as one natural population based on the geographic information on the sampling point. Prior to the seed collection, the latitude, longitude, and elevation of each sampling site were identified using a hand-held global positioning system. Afterward, three quadrats (5 m × 1 0 m) were randomly set at each population. Plenty of seeds of healthy and mature shrub individuals of *A. centraliasiaticus* were collected at each quadrat and mixed together. Then, they were put into cloth bags, labeled, and taken back to the laboratory. After removing crown hairs, seeds were air-dried for one month in the laboratory at room temperature and weeded out immature, incomplete, and insect-infested ones. When achieved to a constant mass, partial seeds were randomly selected for subsequent experiments. The remaining ones were stored dry at the Key Laboratory of Superior Forage Germplasm on the Qinghai-Tibetan Plateau, Xining, Qinghai, China (elevation: 2,276 m a.s.l., without temperature regulation) at a temperature similar to the local environment.

### 2.3 Soil properties and climate data

Three soil samples were collected from 0 to 30 cm depth in each quadrat. Then, they were air-dried in a shaded and ventilated environment and ground and sieved through a 100-mesh sieve before chemical analyses. Analyses of SOC, AN, AP, and pH were carried out using the potassium dichromate titration method, alkaline hydrolysis diffusion method, molybdenum antimony colorimetric method, and potentiometric method, respectively. Climate data, such as MAT and MAP, were extracted from the worldclim database (http://www.worldclim.org/, version 2.1) using ArcGIS 10.8.

### 2.4 Seed morphology and nutrient traits

For each seed sample, 20 representative seeds were selected at random. Then, they were observed and photographed using a Nikon (SMZ18, MODEL P2-FIRL) microscope. The length and width were measured using NIS-Elements D (4.40) software. The length-to-width ratio (LWR) reflecting seed shape was calculated. The thousand-grain weight (TGW) of each seed sample was determined by weighing 1,000 seeds with an electronic balance.

To identify physicochemical properties, a subsample of seeds was milled and oven-dried at 60°C for 72 h to a constant mass. Then, the seeds were ground into powders using a ball mill (model: CBGT-48, CeBo, shanghai, China) to determine nutrient content. The values of the CSP, CSS, CS were measured using a Plant Soluble Protein Extraction Kit (C500071, Sangon Biotech, Shanghai, China), a Plant Soluble Sugar Content Assay Kit (D799393, Sangon Biotech, Shanghai, China), and a Starch Content Assay Kit (D799326, Sangon Biotech, Shanghai, China) according to the manufacturer’s protocols, respectively. CCF was determined using the Soxhlet extraction method (with petroleum ether as a solvent).

### 2.5 Seed germination stress tolerance tests

The climate and soil of the Qinghai-Tibetan Plateau have prominent characteristics, including low MAT, highly variable MAP, large arid regions, and salinized land. Therefore, three completely randomized experiments were conducted to compare the stress tolerance capabilities of *A. centraliasiaticus* during seed germination at multiple sampling sites on the Qinghai-Tibetan Plateau. According to the related literature, the stress tolerance regime during germination was chosen to encompass climatic and environmental changes of the entire collection area during the germination period of seeds.

1) Experiment 1 was to evaluate the drought tolerance capacity of seeds during germination, including water potential treatments at 0 MPa (W1), −0.3 MPa (W2), −0.6 MPa (W3), −0.9 MPa (W4), and −1.2 MPa (W5) to simulate different concentrations of polymer penetrant polyethylene glycol (PEG-6000) solution.
2) Experiment 2 aimed to assess the low-temperature tolerance capacity of each seed sample, containing temperature treatments at 25°C (T1), 20°C (T2), 15°C (T3), 10°C (T4), and 5°C (T5).
3) Experiment 3 consisted of saline-alkali treatments to determine saline-alkali tolerance capacity, including 0 mmol/L (S1), 50 mmol/L (S2), 100 mmol/L (S3), 150 mmol/L (S4), and 200 mmol/L (S5) to simulate different concentrations of saline-alkali solution (the ratio of NaCl: Na2SO4: Na2CO3: NaHCO3 was 9:9:1:1).

The three stress tolerance tests of seed germination were conducted simultaneously in three illuminated incubators (ZRQ-250, Shanghai, China) at 60% relative humidity, 12/12-hour photoperiod, and 5000 lux light intensity from October to November 2023. The temperatures in Exp. 2 and Exp. 3 were specially set to the standard 25°C. Before the test, seeds were immersed in a 5% NaClO solution for 10 minutes for surface sterilization and washed with distilled water four to five times. Then, their surface water was dried with filter papers. Subsequently, the seeds were evenly placed on filter papers laying flat in 9-cm-diameter Petri dishes. The dishes were added in 5ml of solution (Exp. 1: the deionized water; Exp. 2: the PEG-6000 solution; Exp. 3: the saline-alkali solution of varying concentrations), respectively. Each dish contained 50 undamaged seeds randomly selected from each quadrat, and each treatment had three replicates.

The tests lasted for 15 days and were terminated when no newly germinated seeds were observed over five consecutive days. The criterion for germination was a radicle of at least 1 mm long emerged from the seed coat. During testing, pipettes were used for daily renewal of solutions in Petri dishes, and germinated seeds were counted.

To judge the stress tolerance capabilities of seed germination, this study incorporated the concept of seed germination drought tolerance index (GDTI) proposed by Liu et al. (2018) and extended it to the seed germination cold tolerance index (GCTI) and germination saline-alkali tolerance index(GSTI). This study used the germination index (GI) and assigned a weighted value to each treatment in each experiment. Taking T1 (25°C) in Exp. 1, W1 (0 MPa) in Exp. 2, and S1 (0 mmol/L) in Exp. 3 as standard controls, their coefficients were set to the constant 1. The values of GDTI, GCTI, and GSTI were calculated according to the following formulas (1-7)


(1) $${\text{GI}}_{\text{i}}=\sum \frac{{\text{n}}_{\text{i}}}{{\text{t}}_{\text{i}}}$$

(2) $${\text{D}}_{\text{Ri}}=(1+\left|{\text{W}}_{\text{i}}\right|)$$

(3) $${\text{C}}_{\text{Ri}}=\frac{{\text{T}}_{1}}{{\text{T}}_{\text{i}}}$$

(4) $${\text{S}}_{\text{Ri}}=\frac{{\text{S}}_{\text{i}}}{10}\;=\;(2∼5)$$

(5) $$\text{GDTI}=\frac{\sum_{\text{i}=1}^{5}{\text{GI}}_{\text{i}}\times {\text{D}}_{\text{Ri}}}{5}$$

(6) $$\text{GCTI}=\frac{\sum_{\text{i}=1}^{5}{\text{GI}}_{\text{i}}\times {\text{C}}_{\text{Ri}}}{5}$$

(7) $$\text{GSTI}=\frac{{\text{S}}_{\text{R}1}(\sum_{\text{i}=2}^{5}{\text{GI}}_{\text{i}}\times {\text{S}}_{\text{Ri}})}{5}$$

Where *n_i_* is the germination number on the day (*i*); *t_i_* is the corresponding number of days of germination; GI*i* is the germination index; *D_Ri_, C_Ri_*, *and S_Ri_* are the coefficients of the weighted assignments to the treatments in Exp.1, Exp.2, and Exp.3, respectively (*D_R1 =_* 1*, C_R1 =_* 1*, and S_R1 =_* 1); *W_i_*, *T_i_*, and *S_i_* are the values of treatment temperature, water potential, and concentration for the each treatment in Exp.1, Exp.2, and Exp.3, respectively.

### 2.6 Data analysis

The data in this study were collated using Microsoft Excel (Excel 2010, Microsoft, Redmond, WA). Origin 9.8 (Origin Lab Co., Northampton, MA, USA) was employed for plotting. The one-way analysis of variance (ANOVA) was conducted using SPSS 27.0 (SPSS 27.0 for Windows, SPSS Inc., Chicago) to test the significance of seed trait variations among populations with a significance level of *p*< 0.05. This study adopted the maximum value, minimum value, Max/Min (the maximum value divided by the minimum value), mean value, and the coefficient of variation (CV, the standard deviation divided by the mean) to describe seed trait variations among the 21 natural populations. In addition, linear regressions were performed to explore seed morphological traits (seed length, width, LWR, and TGW), nutrient content (CSP, CSS, CS, and CCF), and seed germination stress tolerance indexes (GDTI, GCTI, and GSTI) varying with geographic gradients (latitude, longitude, and altitude).

Multiple regressions were employed to analyze the relationship between environmental variables (climate and soil nutrients) and seed traits (seed morphology, seed nutrients, and germination stress tolerance indexes). Three linear mixed-effect models using the “lmer” function in the R package “lmerTest” (Kuznetsova et al., 2013) were used to determine the effects of seed intrinsic attributes (seed morphology and seed nutrients) on germination stress tolerance indexes (GDTI, GCTI, and GSTI). The fixed effects were seed length, width, LWR, TGW, CSP, CSS, CS, and CCF. The populations were computed as random effects. Before statistical analyses, the normality of all variables was examined using the Shapiro-Wilk test. Three linear mixed-effect models were performed with the statistical program R version 4.3.2 (R Development Core Team, 2023; http://www.r-project.org/).

Structural equation modeling (SEM) was adopted to detect direct and indirect effects of environmental variables (climate and soil nutrients) and seed intrinsic attributes (seed morphology and seed nutrients) on germination stress tolerance indexes (GDTI, GCTI, and GSTI). Climate (MAT and MAP), soil factors (SOC, AN, AP, and PH), seed morphology (length, width, LWR, and TGW), and seed nutrients (CSP, CSS, CS, and CCF) were represented by the first component of Principal Component Analysis (PCA). According to the Chi-square (χ2) value, goodness of fit index (GFI), comparative fit index (CFI), and root mean square error of approximation (RMSEA), the fitting model of SEM was evaluated. SEM was performed using the maximum likelihood estimation method with AMOS 26.0 software (Amos Development Corporation, Chicago, U.S.A.).

## 3 Results

### 3.1 Seed trait variations

The ANOVA results exhibit that all seed traits (seed morphology, seed nutrients, and seed germination stress tolerance indexes) significantly differ (*P*< 0.001) among the 21 populations of *A. centraliasiaticus* on the Qinghai-Tibetan Plateau (**Table 1**). Generally, the magnitude of variations in seed germination stress tolerance indexes is much greater than that in seed morphology and seed nutrients. The latter two are almost identical, with their CVs ranging from 11 to 24. In terms of seed morphology, TGD has the greatest variation, with a maximum record of 0.76 g, a minimum value of 0.30 g, and a CV value of up to 23.01%. Particularly, LWR has a CV value of only 11.06%, the least variation among all seed traits. Regarding seed nutrients, the variation in CSS is particularly prominent, with a CV value of up to 23.94%, almost twice that of the other indicators (CSP, CS, and CCF). Although the variations in germination stress tolerance indexes are significant, the CV values of GDTI, GCTI, and GSTI are nearly identical.

**Table 1:** Seed trait variations of the 21 populations of *Asterothamnus centraliasiaticus* on the Qinghai-Tibetan Plateau.

Seed trait	Minimum	Maximum	Max/Min	Mean	CV (%)	df	F	*P*
Length (mm)	2.05	4.49	2.19	3.31	15.83	20	97.32	< 0.001
Width (mm)	0.70	1.30	1.85	1.00	11.63	20	32.39	< 0.001
LWR	2.22	4.52	2.04	3.29	11.06	20	23.99	< 0.001
TGW (g)	0.30	0.76	2.53	0.55	23.01	20	44.75	< 0.001
CSP (mg/g)	20.17	33.24	1.65	28.16	12.88	20	63.08	< 0.001
CSS (mg/g)	20.12	49.24	2.45	28.45	23.94	20	62.97	< 0.001
CS (mg/g)	38.46	65.21	1.70	49.12	11.50	20	64.02	< 0.001
CCF (%)	0.14	0.30	2.14	0.21	17.94	20	16.41	< 0.001
GDTI	0.23	8.96	38.96	3.89	66.53	20	242.24	< 0.001
GCTI	1.10	39.78	36.16	17.11	64.79	20	278.89	< 0.001
GSTI	6.84	7.71	1.13	7.18	62.87	20	124.94	< 0.001

Analyses were derived from one-way ANOVA; CV, coefficient of variation; LWR, length-to-width ratio; TGW, thousand-grain weight; CSP, content of soluble protein; CSS, content of soluble sugar; CS, content of starch; CCF, content of crude fat; GDTI, germination drought tolerance index; GCTI, germination cold tolerance index; GSTI, germination saline-alkali tolerance index; the same below.

### 3.2 Seed trait patterns along geographic gradients

**Figure 2:**
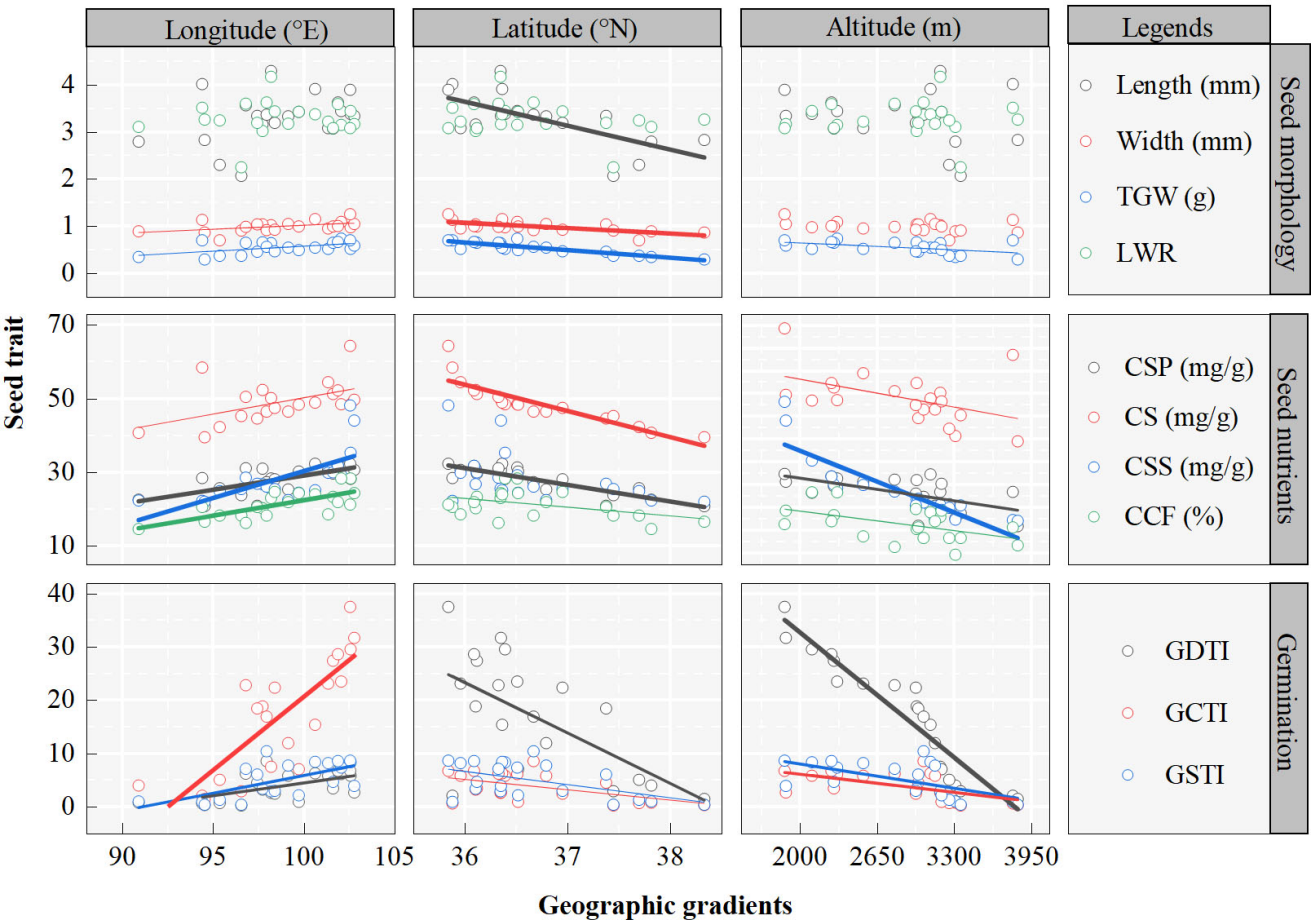
Seed trait patterns along geographic gradients of the 21 populations of *Asterothamnus centraliasiaticus* across its geographic distribution on the Qinghai-Tibetan Plateau. Analyses were derived from linear regressions; The goodness of fit is represented by R^2^. R^2^ and P values are detailed in **Table A2**. The width of the fitting line was determined by the significance of the fit, being thinnest when P<0.05 next to P< 0.01 and thickest at P< 0.001; TGW, thousand -grain weight; LWR, length-to-width ratio; CSP, contents of soluble protein; CSS, contents of soluble sugar; CS, contents of starch; CCF, contents of crude fat; GDTI, germination drought tolerance index; GCTI, germination cold tolerance index; GSTI, germination saline-alkali tolerance index.

The seed traits of *A. centraliasiaticus* on the Qinghai-Tibetan Plateau show regular changes with geographical gradients (**Figure 2**; **Table A2**). All seed traits are significantly correlated with longitude except for length and LWR, and the increase trends with longitude (**Figure 2**). Among them, CSP, CSS, CCF, and GCTI are highly correlated (*P*< 0.001) with longitude, GDTI and GSTI are significantly correlated (*P*< 0.01) with longitude, and width and TGW are correlated (*P*< 0.05) with longitude. Conversely, seed traits are negatively correlated with latitude (the higher the latitude, the lower the seed morphology, nutrient content, and seed germination stress tolerance indexes) (**Figure 2**). All indicators of seed morphology except LWR present highly significant correlations (*P*< 0.001) with latitude. Among seed nutrient traits, CS and CSP have highly significant correlations (*P*< 0.001) with latitude, while CCF, GDTI, GCTI, and GSTI are only significantly correlated (*P*< 0.05) with latitude. In terms of latitude, all seed traits are negatively correlated with altitudinal gradient (**Figure 2**). All seed nutrient traits and germination stress tolerance indexes are significantly correlated (*P*< 0.05) with altitude. CCF and GDTI are significantly correlated (*P*< 0.001) with altitude. TGW is the only trait of seed morphology significantly correlated (*P*< 0.05) with altitude.

### 3.3 Climatic and edaphic effects on seed traits

**Figure 3:**
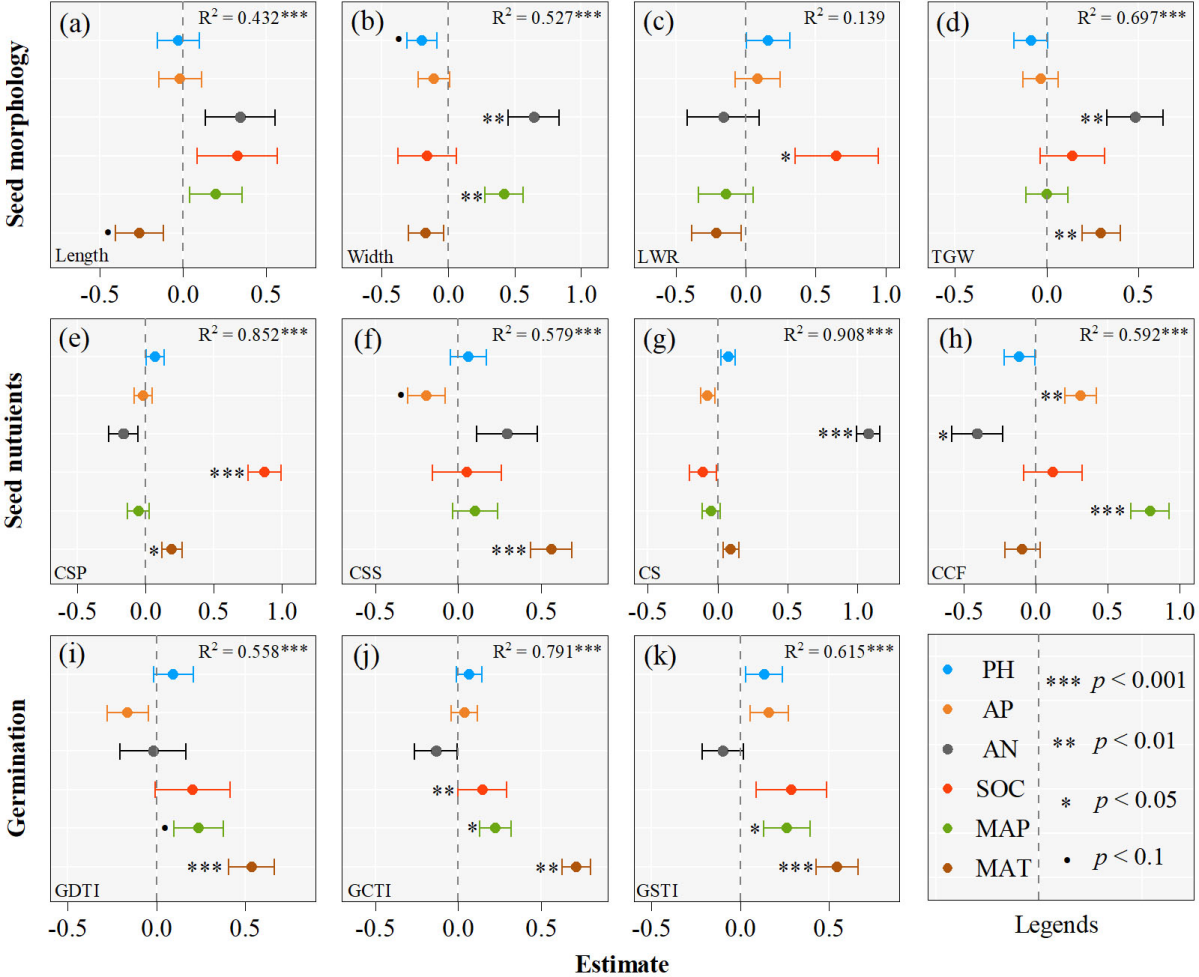
Effects of climate variables and soil nutrients on the seed traits of *Asterothamnus centraliasiaticus* on the Qinghai-Tibetan Plateau. Analyses were derived from multiple regressions; *P*< 0.1; ^*^ *P*< 0.05; ^**^ *P*< 0.01; ^***^ *P*< 0.001; LWR, length-to-width ratio; TGW, thousand -grain weight; CSP, contents of soluble protein; CSS, contents of soluble sugar; CS, contents of starch; CCF, contents of crude fat; GDTI, germination drought tolerance index; GCTI, germination cold tolerance index; GSTI, germination saline-alkali tolerance index.

The results of multiple regression models unequivocally suggest that climatic and edaphic variables can explain different proportions (1.4 - 90.8%) of seed trait variations (**Figure 3**). More than 40% of variations of most seed traits (except for LWR) are explained by climatic and edaphic variables, while only 1.4% of LWR variation is explained (**Figure 3**). Climatic and edaphic variables explain 43 - 70% of the variations in seed morphological traits (except for LWR), more than 60% of the variations in all seed nutrient traits, up to 91% of CS variation, and 56 - 79% of the variations in seed germination stress tolerance indexes.

Key climatic and edaphic variables affecting seed morphological traits, nutritional traits, and seed germination stress tolerance indexes vary among the 21 populations of *A. centraliasiaticus* (**Figure 3**). Regarding seed morphological traits, MAP and AN significantly impact seed width, and MAT and AN are the strongest predictors of TGW. The same relationships also exist between seed length and MAT and between LWR and SOC. Among seed nutrient traits, CSP, CSS, CS, and CCF are most strongly influenced by SOC, MAT, AN, and MAP, respectively. The impacts of MAP and AP on CSP and CS are confirmed. CCF is not only affected by MAP but also by AP and AN. In addition, all the variations in seed germination stress tolerance indexes are attributed to climatic variables (MAT and MAP), barring the effect of SOC on GCTI.

### 3.4 Relationships between seed germination stress tolerance indexes and seed intrinsic attributes

**Figure 4:**
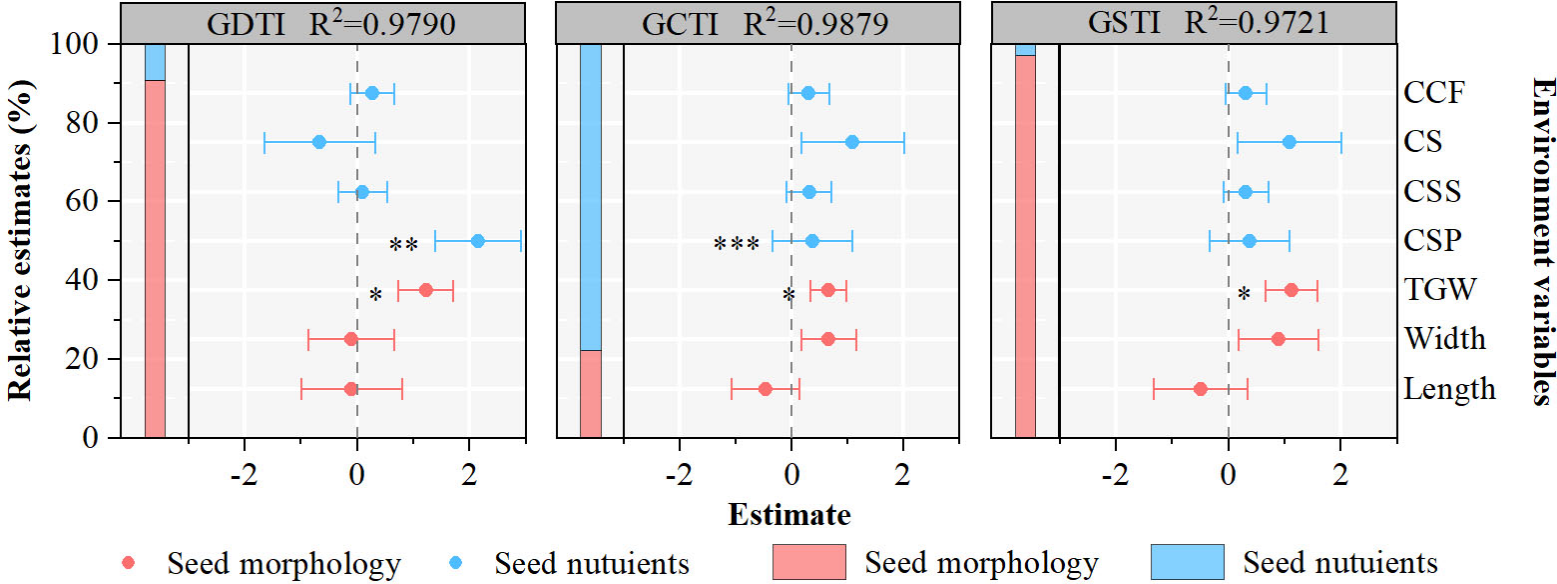
Effects of seed morphology and seed nutrients on the seed stress tolerance germination indexes of the 21 populations of *Asterothamnus centraliasiaticus*. Analyses were derived from The linear mixed-effect models; ^*^ *P*< 0.05; ^**^ *P*< 0.01; ^***^ *P*< 0.001; CCF, contents of crude fat; CS, contents of starch; CSS, contents of soluble sugar; CSP, contents of soluble protein; TGW, thousand -grain weight; GDTI, germination drought tolerance index; GCTI, germination cold tolerance index; GSTI, germination saline-alkali tolerance index.

The linear mixed-effect model, including seed morphology and seed nutrient variables, indicates significant relationships between seed germination stress tolerance indexes and seed intrinsic attributes (**Figure 4**). Through effect decomposition, seed morphology explains more than 90% of GDTI and GSTI. GCTI is influenced by seed morphology and seed nutrients, accounting for 22% and 73%, respectively. Primary seed morphology and seed nutrient variables affecting seed germination stress tolerance indexes vary in the 21 populations of *A. centraliasiaticus* (**Figure 4**). Among them, TGW is the only indicator of seed morphology that affects seed germination stress tolerance indexes and the only seed intrinsic attribute influencing GSTI. CSP is the only seed nutrient parameter that significantly impacts GDTI and GCTI.

### 3.5 Direct and indirect influencing factors of seed trait variations

**Figure 5:**
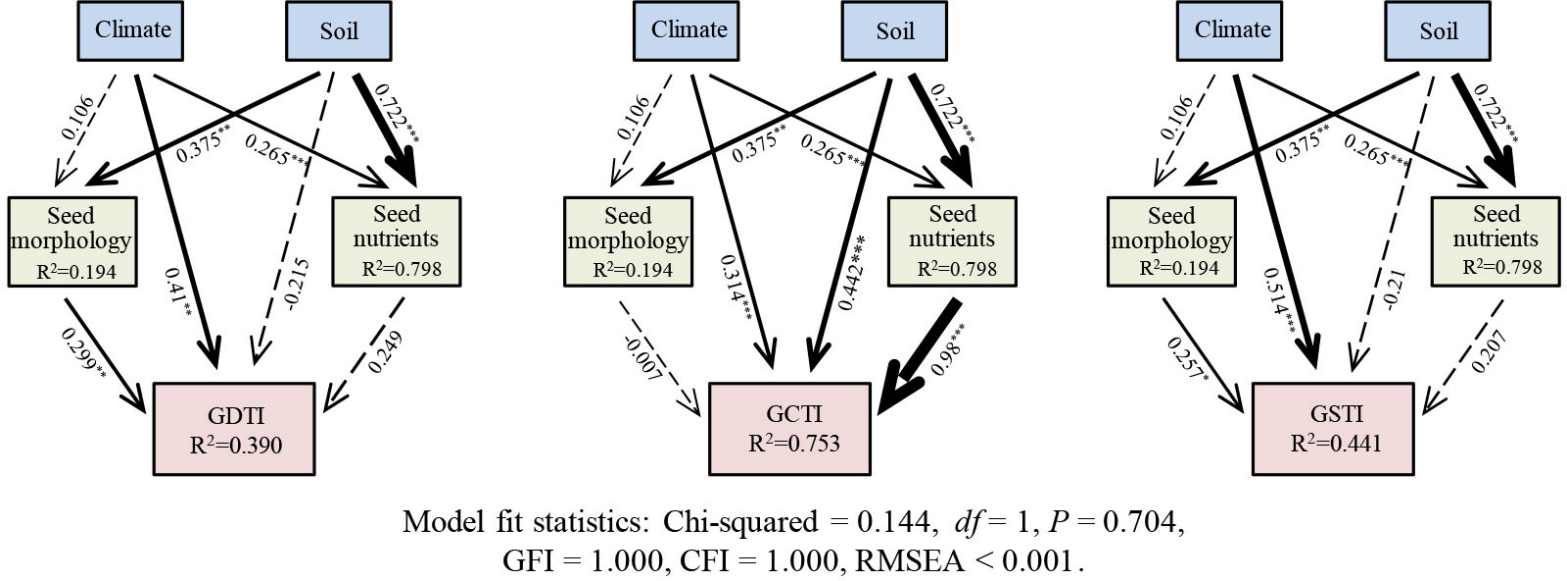
SEM diagram of the direct and indirect influencing factors of seed trait variations of the 21 populations of *Asterothamnus centraliasiaticus* on the Qinghai-Tibetan Plateau. Numbers adjacent to the arrows represent standardized path coefficients. Arrow widths are proportional to standardized path coefficients. The portion of the variance explained by the model is indicated by R^2^. Solid lines denote significant effects, and dotted lines represent non-significant effects; GDTI, germination drought tolerance index; GCTI, germination cold tolerance index; GSTI, germination saline-alkali tolerance index; *P< 0.05; **P< 0.01; ***P< 0.001.

SEM shows that the climate, soil nutrients, seed morphology, and seed nutrients together explain 39.0%, 75.3%, and 44.1% of the variations in the GDTI GCTI, and GSTI of the 21 populations of *A. centraliasiaticus* (**Figure 5**), respectively. Climate has no direct effect on seed morphology but significantly affects seed nutrients. Soil nutrients significantly and directly impact seed morphology and seed nutrients. Furthermore, climate and seed morphology directly influence GDTI and GSTI, while soil nutrients indirectly affect them by influencing seed morphology. GCTI is not only directly affected by climate, soil nutrients, and seed nutrients but also indirectly impacted by climate and soil nutrients by influencing seed nutrients.

## 4 Discussion

### 4.1 Seed trait patterns and geographic gradients

The seed traits of *A. centraliasiaticus* vary substantially among the 21 natural populations, and most seed traits show significant geographic patterns, indicating that variations in seed traits are not only influenced by natural selection but also by adaptation to mutable environments. In addition, due to the unique geographical location and climate of the Qinghai-Tibetan Plateau, climatic factors (MAT and MAP) are highly correlated with its latitude, longitude, and altitude. Therefore, it is suggested that there is an overlap in the variations in seed traits with latitude, longitude, and elevation (Wang et al., 2021). This is aptly confirmed by the findings in this study that almost all seed traits vary significantly with geographical gradients, increasing with latitudinal gradient and decreasing with longitudinal and altitudinal gradients. It also demonstrates inversely that as an ecoregion of global concern, the Qinghai-Tibetan Plateau has inherent climate change regularities that make it perfect for biological adaptation research.

Previous studies have proven that most plant seed traits decrease from south to north along latitudinal gradient (Pieter et al., 2013). It implies that at low latitudes, where the environment is usually warmer and wetter, and plants face more competition, seeds tend to be smaller, which favors germination and growth in a competitive but suitable environment (Murray et al., 2004). At high latitudes, plants need more seed reserves for nutrients and faster germination due to the short season and harsh environmental conditions. Therefore, their seeds are usually larger and have a higher germination percentage (Moles et al., 2007). However, in this study, seed morphology, seed nutrients, and germination stress tolerance capabilities increase with altitudinal gradient. It is surmised that this is due to the specificity of the study area. Variations in seed traits across latitudinal gradient are not only the result of plant adaptation to diverse environmental conditions but are also directly affected by environmental conditions. Environmental conditions are more severe at higher latitudes, which may lead to greater variations in plant populations. Conversely, environmental conditions at lower latitudes may cause genotypes to adapt to fewer variations. Moreover, the complex and variable microenvironment of the Qinghai-Tibetan Plateau is a major contributor to this result. It is found that MAT tends to increase with latitude in this study area, and temperature is the main determinant of the distribution of most shrub species in this region (Li et al., 2023).

Along the longitudinal gradient, seed traits decrease from west to east, with the most significant changes in seed morphological traits. The same findings were confirmed by Meng et al. (2017) and Wu et al. (2018), who suggested that variations in plant seed traits with longitude were closely related to local climate. The results of this study evidence that geographic patterns of seed traits are connected with the local environment. For instance, seed width decreases from west to east under the significant effect of MAP (**Figure 3**). The incremental effect of humidity from the ocean to the inland is not considered a valid explanation for the results of this study. Because the special geographical position of the Qinghai-Tibet plateau and its complex topography in the region dilute this effect. It should be complemented by other reasons, such as the research area (the Qinghai-Tibetan Plateau), which exhibits a remarkable decrease in temperature and increases in precipitation from west to east and in altitude.

Generally, the harsh and unpredictable environments at high elevations may impose a rigorous filter on seed traits (Wang et al., 2021). In this study, most seed traits show a decreasing trend with gradients. It is consistent with Qi et al. (2014) and Wang et al. (2021) that morphological traits decrease at high elevations, especially seed mass (TGW in this study). The higher the altitude, the shorter the growing season for plants and the greater the environmental stress (Angela and Mark, 2004; Körner, 2007). The extreme environmental conditions can abate the availability of plant resources, thereby lowering the allocation of resources to individual seeds. Under such conditions, the distributional trade-off between seed size and seed number may produce smaller seeds, which could be a bet-hedging strategy for plants in uncertain environments (Wang et al., 2021). But there are also studies claiming that plants also select large seeds with high survival probabilities in extreme environments (Wu et al., 2022).

In addition, this study found that seed nutrients and seed germination stress tolerance capabilities indexes were significantly negatively correlated with altitude gradient. It is speculated that this phenomenon is closely linked to insufficient soil nutrients at high altitudes. The higher the altitude, the poorer the soil nutrients, the fewer nutrients are available to the plant, and the fewer resources are passed on to the next generation, resulting in lower seed nutrient content and weakened seed germination stress tolerance capabilities.

### 4.2 The effects of climate and soil variables on seed trait variations

Climate and soil nutrients are two primary categories from an environmental perspective. Plants of the same genotype in different geographical locations may generate seeds with various morphology and nutrient content (Snider et al., 2016; Reguera et al., 2018). Similarly, the seeds of the same plant may vary every year (McGraw et al., 1986). Undoubtedly, the effects of these sites and years on seed traits are due to differences in the environmental conditions experienced by the parent plant during seed development.

In this study, climatic factors (MAT and MAP) positively influence seed traits, perhaps because high temperature is beneficial to photosynthesis and can promote the biomass of seeds (Wiegand and Cuellar, 1981). Another possible cause is that temperature and precipitation affect a range of enzyme and transporter protein activities in parent plants and seeds (Sehgal et al., 2018). Additionally, this study found a stronger response of seed nutrients to climate than seed morphology. This could be attributed to the effects of temperature and precipitation on seed nutrient accumulation by influencing starch and protein synthesis processes, directly leading to variability in seed nutrients and further affecting seed morphology and seed germination stress tolerance capabilities (Behboudian et al., 2001; Asthir et al., 2012).

Soil factors in the environment of the parent plant, such as SOC, AN, AP, and pH, can cause changes in seed traits (Wu et al., 2018). Therefore, soil nutrients are another major factor in the variations of seed traits (Sun et al., 2012). This experiment observed that the effects of soil nutrients on seed morphology and seed nutrients reached significant levels, compared to seed morphology a stronger effect on seed nutrients. This may be because seed morphology is the least plastic of plant traits, and seed nutrients are more intuitive in their response to soil nutrients than seed phenotype. Among these soil nutrient factors, the effect of soil PH on seed traits is not observed. The reason may be that due to long-term saline adaptation, all the populations of *A. centraliasiaticus* in the study area have synergistically accomplished the adaptation to the saline soil environment. In addition, SOC, AN, and AP significantly affect seed phenology and seed nutrients. Among them, soil AN is significantly correlated with CS and CCF and is a major cause of the variability in seed nutrient content (Malhi et al., 2014; Huang et al., 2016). The AN content of the soil in which the mother plant survives significantly affects the amount of amino acids in the seeds, with a consequent overall increasing effect on seed protein content (Zhang et al., 2016). In addition, the high nitrogen content of the soil leads to a decrease in the seed fat content, and resulting in an opposite trend in the protein and fat content of the seeds (Solis et al., 2013). Moreover, this experiment also found that soil AN content caused opposite trends in seed CS and CCF. This may be due to the competition between fatty acids and amino acids for the carbon skeleton produced by carbohydrate metabolism during seed nutrient synthesis. Whereas high soil nitrogen promotes nitrogen metabolism in the seeds and accelerates protein and starch synthesis (Arduini et al., 2006), it conversely limits fatty acid synthesis, leading to a reduction in seed fat content (Rathke et al., 2005).

### 4.3 Direct and indirect influencing factors of seed germination stress tolerance capabilities

In general, differences in seed germination stress tolerance capabilities may be the result of interaction between the seeds and environmental conditions. This study found that regarding climate and soil nutrients, climate had a highly significant effect on the GDTI, GCTI, and GSTI of seeds. Soil nutrients affected GCTI directly, and their direct effects on GDTI and GSTI were weaker. Existing literature may shed light on this phenomenon. Meyer et al. (1995) showed that germination strategies were habitat-specific among populations. One possible explanation is that a harsher climate tends to guide plant seed variations toward a higher germination percentage. It agrees with the life-history strategy of r-strategist (an organism that has the ability to reproduce and spread rapidly in an ecosystem, usually have a high growth rate and low competitiveness), which can lead to the settlement of small seeds after rapid germination (Souza and Fagundes, 2014). The effect of soil nutrients on seed GCTI may be due to the high levels of soil SOC, providing more material and energy reserves to plants and seeds. We can further speculated that drought and salinity are essentially water stresses on plant seeds that are directly driven by climate, and plant may be responded to by an increase in plant morphology, which further promotes seed swelling. Cold is unrelated to plant seed morphology and can only be responded to by the soil providing more nutrients to the plant, which are then passed on to the seed to ensure germination.

The results of this study shows that seed morphology explains substantially more GDTI and GSTI variations than seed nutrients (**Figure 4**), indicating that seed morphology is the major direct influencing factor of seed germination drought and saline-alkali tolerance capabilities. TGW is the most significant influencing factor, which aligns with other studies (Hantsch et al., 2013; Wu et al., 2018). This can be interpreted as large seeds reserve higher resources and provide more nutrients during the process of seed-seedling transition (Amonum et al., 2020). Meanwhile, it is detected that seed CSP positively affects seed germination drought and cold tolerance, representing that seed nutrient concentrations can influence plant recruitment (Yu et al., 2017). This agrees with previous findings that seeds with higher nitrogen concentrations perform better under harsh environmental conditions (Naegle et al., 2005). A more plausible explanation is that soluble proteins act as enzymes, which can promote the metabolism of substances. An elevation in their content is an important indicator of the increasing vital activity of the embryo (Singkhornart and Ryu, 2011). The higher the protein activity within the seed, the richer the metabolic activity and the higher the seed germination vigor (To et al., 2002). In summary, the joint analysis of phenotypic, physiological, and molecular levels in follow-up studies may provide some inspiration for exploring the mechanisms of seed germination stress tolerance capabilities. Although this is out of the scope of the current study, it is expected to be included in future work.

## 5 Conclusions

This study found substantial intraspecific variations in the seed traits of the 21 populations of *A. centraliasiaticus* on the Qinghai-Tibetan Plateau. Most seed traits, except for LWR, vary significantly along geographic gradients. In addition, this study confirmed the direct and indirect effects of climatic (MAT and MAP) and soil nutrient (SOC, AN, AP, and PH) variables on seed traits. The effect of climate on seed traits is stronger on seed nutrients, while soil nutrients significantly affect seed morphology and seed nutrients. Climate directly drives variations in GDTI and GSTI, while GCTI is influenced by climate and soil nutrients (mainly SOC). Meanwhile, among seed intrinsic attributes, the variations in GDTI and GSTI receive more influence from seed morphology (mostly TGW). Seed nutrients (largely CSP) significantly impact GCTI variation. This study provides a detailed explanation of seed trait variation patterns in shrubs in alpine desert ecosystems through studying one representative species, which have important implications for understanding the mechanisms of shrub adaptation in alpine desert ecosystems, predicting the outcomes of environmental change, and informing conservation efforts. Moreover, this study makes a valuable contribution to the management of alpine desert ecosystems on the Qinghai-Tibetan Plateau.

## Data availability statement

The original contributions presented in the study are included in the article/**Supplementary Material**. Further inquiries can be directed to the corresponding authors.

## Ethics statement 

According to No. 221(E) Article 15: (1)-ii) of the International Union for the Protection of New Varieties of Plants (UPOV) in 1991, this study did not necessitate ethical approval or permission. Similarly, research on plant seed does not require ethical approval.

## Author contributions

ZSL: Conceptualization, Data curation, Formal analysis, Investigation, Methodology, Resources, Software, Visualization, Writing – original draft, Writing – review & editing. YSM: Conceptualization, Formal analysis, Funding acquisition, Investigation, Resources, Supervision, Writing – review & editing. YL: Conceptualization, Formal analysis, Investigation, Methodology, Resources, Writing – review & editing. YLW: Conceptualization, Data curation, Formal analysis, Investigation, Methodology, Project administration, Resources, Supervision, Visualization, Writing – review & editing. XYW: Data curation, Formal analysis, Investigation, Methodology, Project administration, Software, Validation, Writing – original draft, Writing – review & editing.
