# Supplementary material for: Geographical patterns and environmental influencing factors of variations in Asterothamnus centraliasiaticus seed traits on Qinghai-Tibetan plateau
Source: Front Plant Sci. 2024 Mar 28;15:1366512. doi: 10.3389/fpls.2024.1366512 (PMC11006976; doi:10.3389/fpls.2024.1366512)
Supplement: Supplementary file 1 [file Table_1.docx]

**Appendices**

Table A1. Site characteristics of the 21 populations of *Asterothamnus centraliasiaticus* across its geographic distribution on the Qinghai-Tibetan Plateau.

| Site code | Latitude (°N) | Longtitude (°N) | Altitude (m) | MAP (mm) | MAT (℃) | Location information |
| --- | --- | --- | --- | --- | --- | --- |
| GS | 37.8089 | 90.857 | 3307 | 67 | 0.26 | Gasi town, Mangya |
| XTS | 37.6915 | 95.3348 | 3253 | 78 | 1.8 | Xitieshan town, Dachaidan |
| BL-3 | 36.3204 | 96.762 | 2793 | 80 | 4.78 | Balong town, Doulan |
| MZ | 38.3256 | 94.5097 | 3832 | 88 | -1.54 | Minzhu town, Dachaidan |
| GLMD | 35.8716 | 94.3538 | 3791 | 118 | 0.15 | Guolemude town, Geermu |
| HTTL | 37.4389 | 96.5245 | 3351 | 125 | 2.5 | Huaitoutala town, Delingha |
| XRD | 36.101 | 97.687 | 2978 | 157 | 4.47 | Xiangride town, Doulan |
| ZWL | 37.3678 | 97.3986 | 2994 | 171 | 4.03 | Zongwulong town, Delingha |
| BL-2 | 36.6634 | 97.9113 | 3038 | 180 | 3.33 | Balong town, Doulan |
| BL-1 | 36.3402 | 98.1531 | 3177 | 200 | 3.04 | Balong town, Doulan |
| KK | 36.9425 | 98.3688 | 2971 | 202 | 2.61 | Keke town, Wulan |
| CK | 36.7867 | 99.1073 | 3134 | 229 | 1.21 | Chaka town, Wulan |
| QJ | 36.5122 | 99.6833 | 3188 | 287 | 1.48 | Qieji town, Gonghe |
| SCK | 36.3438 | 102.7602 | 1875 | 335 | 8.02 | Shangchuankou town, Minhe |
| HS | 36.3866 | 102.5392 | 2096 | 359 | 6.81 | Hongshui town, Ledu |
| PA | 36.5034 | 102.0206 | 2309 | 375 | 6.23 | Pingan town, Pingan |
| DB | 36.3559 | 100.5957 | 3095 | 400 | 2.39 | Dongba town Gonghe |
| HD | 36.112 | 101.5886 | 2280 | 404 | 6.62 | Hedong town, Guide |
| QS | 35.8348 | 102.5336 | 1866 | 408 | 8.27 | Qingshui town, Xunhua |
| JJ | 36.082 | 101.8585 | 2260 | 411 | 6.64 | Jiajia town, Jianzha |
| XJ | 35.9536 | 101.3087 | 2528 | 427 | 5.35 | Xiejie town, Guide |

Table A2. *P*-values and R^2^ of the linear fitting for the seed trait variations of the 21 populations of *Asterothamnus centraliasiaticus* on the Qinghai-Tibetan Plateau across geographic gradients.

| Seed trait | | Longitude | |  | Latitude | |  | Altitude | |
| --- | --- | --- | --- | --- | --- | --- | --- | --- | --- |
|  |  | *P* | R^2^ |  | *P* | R^2^ |  | *P* | R^2^ |
| Seed morphology | Length | 0.1101 | 0.1288 |  | < 0.001 | 0.4472 |  | 0.3478 | 0.0465 |
|  | Width | 0.2692 | 0.2323 |  | < 0.001 | 0.4685 |  | 0.0971 | 0.1312 |
|  | LWR | 0.8246 | 0.0027 |  | 0.2075 | 0.0822 |  | 0.8693 | 0.0015 |
|  | TGW | 0.0105 | 0.2976 |  | < 0.001 | 0.7715 |  | 0.0175 | 0.2627 |
| Seed nutrients | CSP | < 0.001 | 0.4666 |  | < 0.001 | 0.7558 |  | 0.0040 | 0.3604 |
|  | CSS | < 0.001 | 0.4736 |  | 0.0286 | 0.2282 |  | < 0.001 | 0.7578 |
|  | CS | 0.0204 | 0.2520 |  | < 0.001 | 0.7694 |  | 0.0305 | 0.2234 |
|  | CCF | < 0.001 | 0.5453 |  | 0.0411 | 0.2017 |  | 0.0179 | 0.2613 |
| Germination | GDTI | 0.0042 | 0.3575 |  | 0.0171 | 0.2644 |  | < 0.001 | 0.8887 |
|  | GCTI | < 0.001 | 0.6501 |  | 0.0038 | 0.3632 |  | 0.0065 | 0.3293 |
|  | GSTI | 0.0014 | 0.4224 |  | 0.2694 | 0.0159 |  | 0.0037 | 0.3655 |
